# Serotypes With Low Invasive Potential Are Associated With an Impaired Antibody Response in Invasive Pneumococcal Disease

**DOI:** 10.3389/fmicb.2018.02746

**Published:** 2018-11-15

**Authors:** Nils Littorin, Fabian Uddén, Jonas Ahl, Fredrik Resman, Hans-Christian Slotved, Simon Athlin, Kristian Riesbeck

**Affiliations:** ^1^Clinical Microbiology, Department of Translational Medicine, Faculty of Medicine, Lund University, Malmö, Sweden; ^2^Infectious Diseases, Department of Translational Medicine, Faculty of Medicine, Lund University, Malmö, Sweden; ^3^Department of Bacteria, Parasites and Fungi, Statens Serum Institut, Copenhagen, Denmark; ^4^Department of Infectious Diseases, Faculty of Medicine and Health, Örebro University, Örebro, Sweden

**Keywords:** antibody, opsonization, sepsis, serotype, *Streptococcus pneumoniae*

## Abstract

Pneumococcal polysaccharide vaccines may elicit a hyporesponse under certain conditions. There is limited knowledge, however, on the type of specific antibody response in individuals with invasive pneumococcal disease (IPD). The aim of this study was to investigate the functional antibody response in patients with IPD caused by different serotypes. Pre-immune and convalescent sera from 40 patients (age 14–91 years) with IPD caused by serotypes with low (serotype 3, 19F, and 23F) and high (1, 4, 7F, and 14) invasive potential were investigated. For each patient, the homologous serotype-specific antibody concentration was determined. The functionality of induced antibodies post-IPD was evaluated in an opsonophagocytic assay (OPA). Undetectable or decreased pneumococcal killing in OPA following IPD, i.e., a nonfunctional antibody response, was observed in 24 of 40 patients (60%). Patients with nonfunctional antibody responses had lower serotype specific IgG antibody ratios post-IPD than patients with increased OPA titres. A nonfunctional antibody response was associated with low invasive serotypes (3, 19F, and 23F, *p* = 0.015). In conclusion, a nonfunctional antibody response may follow IPD, and was in our cohort associated to serotypes with low invasive potential. These findings need to be confirmed in a larger material.

## Introduction

Despite widespread immunization with pneumococcal conjugate vaccines (PCVs) and effective antimicrobial therapy, *Streptococcus pneumoniae* is still a major cause of upper and lower respiratory tract infection as well as invasive pneumococcal disease (IPD). Pneumococci are generally shielded by an immunogenic polysaccharide capsule determining the specific serotype based upon chemically unique structures. Immunologic hyporesponses associated with serotypes included in PCVs have been reported in some settings; in clinical trials of an 11-valent PCV preparation in children, IgG titers for serotype 3 were lower following the booster dose compared with titres obtained after the initial dose (Poolman and Borrow, 2011). Following PCV7 immunization, a hyporesponse has been demonstrated in the context of prior pneumococcal carriage in the nasopharynx (Dagan et al., 2010a; Väkeväinen et al., 2010; Rodenburg et al., 2011). Pneumovax^®^ (PPV23), a polysaccharide-based vaccine advocated for immunocompromised hosts and individuals >65 years of age, has also been associated with an attenuated antibody response upon revaccination (O’Brien et al., 2007). The explanation for hyporesponses is not fully understood, but depletion of a specific B-cell pool (i.e., clone) has been proposed (Brynjolfsson et al., 2012). In vaccine trials, excessive concentrations of polysaccharide have been found to reduce the antibody response (Dagan et al., 2010b).

Serotypes differ in their ability to cause invasive disease and in prevalence of nasopharyngeal colonization. Brueggemann et al. (2004) studied the invasive disease potential of different *S. pneumoniae* serotypes in children . The authors concluded that some serotypes (including 3, 6B, 15B/C, 19F, and 23F) conveyed a lower risk for invasive disease, and were more frequently isolated as colonizing bacteria than other serotypes (including 1, 4, 7F, 14, and 18C). These results have been confirmed by other groups (Kronenberg et al., 2006; Zemlickova et al., 2010). Low invasive serotypes are associated with higher case-fatality rates and disease in immunocompromised patients, acting as “opportunistic” bacteria, whereas highly invasive serotypes more often infect healthy, immunocompetent individuals, acting as primary pathogens. In parallel, we have previously demonstrated that low invasive *S. pneumoniae* serotypes induced lower IgG titer ratios than did highly invasive serotypes following pneumococcal pneumonia in adults (Athlin et al., 2014). However, patient serum IgG titers alone do not necessarily reflect the functionality of antibodies, and therefore the IgG-dependent capacity to induce opsonization needs to be measured in functional assays.

Studies *in vivo* have revealed impaired B-cell function and antibody production in sepsis survivors, even long after the septic event has resolved (Pötschke et al., 2013; Griffith et al., 2016). However, it is currently unknown whether IPD may induce an impaired antibody response similar to the hyporesponse observed with pneumococcal vaccines. The objective of this explorative study was to investigate the antibody response following IPD in a cohort of IPD patients.

## Results

### Three Different Types of Responses Are Observed in Opsonophagocytosis

To determine whether IPD related to certain pneumococcal serotypes induces a nonfunctional antibody response, patient sera were collected from two different counties in Sweden. Clinical features of all study patients are outlined in Supplementary Material. Patients were diagnosed with pneumonia (38/40 cases), meningitis or ethmoiditis (1 case each). Median age was 59.5 years (range 14–91 years) and 55% (*n* = 22) were women.

**FIGURE 1:**
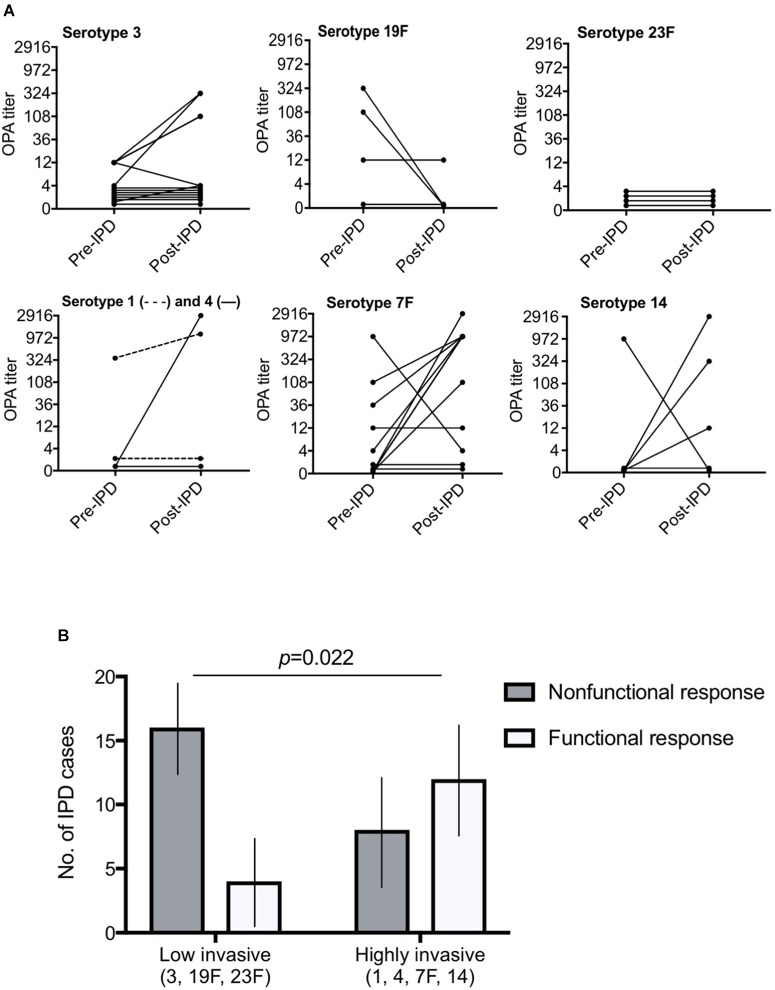
Killing of pneumococci in an OPA revealed mixed responses. Low-invasive *S. pneumoniae* were significantly associated with a nonfunctional response compared to highly invasive serotypes. In **(A)**, 7 different pneumococcal serotypes were analyzed by OPA. The threshold for significant bacterial killing was defined as an OPA titer >1:8. Each line represents pre-IPD and post-IPD sera from one patient. A decreased response was defined by a decreasing curve, whereas an increasing curve and titers above the threshold indicates a functional antibody response. Undetectable titers or titers below the 1:8 titer threshold pre- and post IPD were nonresponsive. **(B)** Low-invasive serotypes (3, 19F, and 23F) were significantly associated with a nonfunctional antibody response (decreased or nonresponsive; Fisher’s exact test *p* = 0.022) compared to highly invasive serotypes (1, 4, 7F, and 14). Invasive potential was defined according to a study by Brueggemann et al. (2004). Black vertical lines represent 95% confidence interval.

We analyzed sera from patients with IPD in a single serotype opsonophagocytic assay (OPA) comprising activated human phagocytes according to a well established protocol. In this functional assay with pre-IPD and post-IPD patient sera, variable OPA responses were observed between serotypes (Figure 1A). According to the antibody responses detected, the IPD patients were categorized either as having a functional antibody response as judged by titers in OPA (*n* = 16), a nonresponse (*n* = 18), or a decreased response (reduced titers in OPA; *n* = 6). The majority of patients (*n* = 24; 60%) thus had a nonfunctional antibody response.

### IPD With a Low-Invasive Serotype Is Associated With a Nonfunctional Response

To examine whether the pneumococcal capsular serotype can be related to a nonfunctional antibody response in our cohort, patients that had suffered from IPD were divided into two groups according to the invasive potential (low invasive or highly invasive) of the infecting pneumococcal serotype according to Brueggemann et al. (2004). Interestingly, a significant difference was observed as outlined in Figure 1B. In total, only 4 of 20 cases (20%) with low invasive serotypes (3, 19F, and 23F) developed a functional antibody response as compared to sera from patients that had been infected with highly invasive pneumococcal serotypes (1, 4, 7F, and 14; *p* = 0.022). Twelve out of 20 cases (60%) developed an increased antibody response in this group.

In a multivariate logistic regression model with functional responses collapsed to a dichotomous response variable (functional antibody response vs. nonfunctional antibody response), infection by a low invasive serotype (3, 19F, and 23F) was the only predictor significantly associated with a nonfunctional antibody response, adjusted for old age and disease severity (*p* = 0.015, Table 1). Old age (>65 years), the Charlsons Comorbidity Index, and disease severity (sepsis or septic shock) were predictors not significantly associated with the results obtained by OPA. Time of sera collection post-IPD was also determined as it might influence the quality or quantity of antibodies, but was not associated to the results in OPA (Tables 1, 2).

**Table 1:** Univariate and multivariate logistic regressions for functional (1) vs. non-functional (0) antibody response (nonresponses and decreased responses collapsed).

Predictor	Univariate odds ratio (95% CI)	*p*	Adjusted odds ratio (95% CI)	*p*
Low-invasive serotype	0.17 (0.04–0.68)	**0.013**	0.13 (0.024–0.67)	**0.015**
Age > 65 years	2.54 (0.63–10.17)	0.188	3.90 (0.70–21.08)	0.119
Sex	0.86 (0.24–3.24)	0.842		
Charlson Comorbidity Index	0.77 (0.57–1.046)	0.095		
Sepsis (severe sepsis or shock vs. sepsis)	0.26 (0.07–1.07)	0.062	0.31 (0.06–1.56)	0.157
Time post-IPD serum (months)	0.98 (0.95–1.02)	0.379		


*Significant p-values (≤0.05) are indicated in bold.*

**Table 2:** Distribution of clinical predictors among individuals with different types of functional responses.

Predictor	Functional antibody response (*n* = 16)	Nonresponse (*n* = 18)	Decreased response (*n* = 6)	*p*-value
Low-invasive serotype, *n* (%)	4 (25%)	14 (68%)	3 (50%)	**0.029**^3^
Age > 65 years	3 (21%)	9 (50%)	3 (38%)	0.263^2^
Sex (% female)	63%	44%	66%	0.827^3^
Charlsons Comorbidity Index, median (IQR)^1^	1 (0–3.0)	4 (1.3–6.0)	2.5 (0.0–4.0)	0.067^2^
Severe sepsis or shock *n* (%)	6 (46%)	14 (78%)	3 (50%)	0.080^3^
Time post-IPD serum in months, median (IQR)	3.5 (1.0–20.0)	12.5 (1.0–38.5)	1.0 (1.0–14.5)	0.377^2^
IgG ratio (post-/pre-IPD), median (IQR)	2.25 (1.8–5.5)	1.78 (0.6–2.6)	0.52 (0.1–0.8)	**0.041**^2^


*Significant p-values (≤0.05) are indicated in bold. ^1^IQR; interquartile range, ^2^Kruskal-Wallis, ^3^Chi-Square test.*

### Serotype Specific IgG Correlates to an Efficient Phagocytic Killing

**FIGURE 2:**
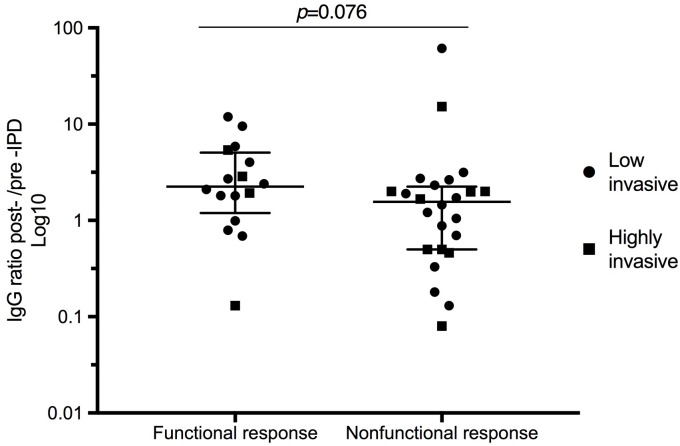
Serotype specific IgG ratios in relation to type of functional antibody response in OPA. Low invasive serotypes are indicated by black dots and highly invasive by squares. Post-IPD IgG antibody concentrations were divided with pre-IPD IgG levels to obtain a ratio for each patient. Median values and interquartile range are indicated. The IgG levels were measured according to a WHO protocol including 007sp reference serum. Patients with a decreased response and a nonresponse were here collapsed as a nonfunctional antibody response in OPA. These patient sera had a lower median serum IgG ratio than patient sera with a functional response.

The concentration of specific anti-capsular IgGs plays an important role for an efficient opsonophagocytosis. IgG levels were therefore determined by ELISA, and the ratio between post- and pre-/acute-IPD IgG titers against capsular antigen of the infecting serotype was calculated for each patient. A higher ratio indicated a stronger increase in anti-capsular antibodies after IPD. As shown in Table 2, sera from patients with a functional response in our OPA had an increased median IgG ratio of 2.25 as compared to 1.78 for individuals with a nonresponse and 0.52 for a decreased antibody response (*p* = 0.041). The same trend was also found when the functional and nonfunctional antibody response (nonresponse and decreased response collapsed) was compared (*p* = 0.076) (Figure 2). However, no difference in IgG titer ratio was observed between patients infected by *S. pneumoniae* highly invasive serotypes (1, 4, 7F, and 14) compared to low invasive serotypes (3, 19F, and 23F, *p* = 0.781).

Five out of 40 convalescent sera, all from patients infected with serotype 3, did not have an IgG concentration considered to be protective for invasive disease (0.35 mg/L) (Siber et al., 2007). All anti-capsular IgG titres are shown in Supplementary Material.

Since IgG or IgG2 deficiencies have been associated with an increased risk for IPD (Ekdahl et al., 1997a; Furst, 2009), we determined total IgG or IgG2 in pre-/acute-IPD sera by ELISA. Values according to local clinical guidelines for measurement of deficiencies in IgG (6.7–15.5 g/L) and IgG2 (1.15–5.7 g/L) were used as reference. All titers were above the lower reference limits of Ig concentrations. In conclusion, a relative increase in specific IgG titers in post-IPD sera was associated with enhanced bacterial killing. Moreover, individuals with a nonfunctional antibody response did not suffer from IgG or IgG2 subclass deficiencies.

## Discussion

We found that a majority of patients (60%) in our cohort developed a nonfunctional antibody response post-IPD. This type of dysfunctional humoral response to IPD seems to be serotype-dependent. Low invasive serotypes (3, 19F, and 23F) were associated with a nonfunctional response in OPA, compared to highly invasive serotypes (serotypes 1, 4, 7F, and 14) that were correlated to a functional opsonophagocytosis. Prior to this study we found that low invasive serotypes were also associated with a poor IgG response in adults with pneumococcal pneumonia (Athlin et al., 2014). The results raise questions on the humoral antibody response in patients that have survived IPD. Our findings should be regarded as exploratory and descriptive (due to a low number of patients), since this study was not designed to test this hypothesis *per se*. Importantly though, in a previous study with fewer IPD cases and flow cytometry analysis as read out instead of OPA, Musher et al. (2000) also revealed that a high percentage (50%) of convalescent sera from bacteremic patients displayed poorly functional opsonization.

The thickness of the polysaccharide capsule is important for the virulence of pneumococcal serotypes. Weinberger et al. (2009) used fluorescein isothiocyanate (FITC)-dextran exclusion to reveal that serotypes 3, 19F, and 23F have thicker capsules compared to 1, 4, 7F, and 14. The nonfunctional antibody response in low invasive serotypes found in the present study may be due to this difference in capsular structure. Some support for this hypothesis can be attained in investigations on PCV:s, where excessive amounts of polysaccharide in vaccine formulas reduced the antibody response (Dagan et al., 2010b). An explanation for the impaired antibody response observed in polysaccharide vaccines was suggested by Brynjolfsson et al. (2012). In autopsies of mice immunized with meningococcal polysaccharides they found increased apoptosis of polysaccharide specific B-cells (Brynjolfsson et al., 2012). Yet another theory was put forward by Poolman and Borrow (2011). They proposed that the hyporesponse observed in relation to immunization with abundantly encapsuled serotype 3 may be due to absorption of residual antibodies upon exposure.

Other hypotheses for nonresponsiveness to pneumococcal polysaccharide capsules have also been suggested. In a study by Simell et al. (2011) aging reduced the functionality of anti-pneumococcal antibodies and consequently killing of *S. pneumoniae* by opsonophagocytosis. In our material, age >65 years was not associated with a nonfunctional antibody response. Cases of nonfunctional antibody responses were observed in several younger patients (for example, patients aged 14–28 years as indicated in Supplementary Material), suggesting that other mechanisms than immunosenescence are involved.

The powerful immunological reaction observed in septic patients is followed by an immune suppressive state that might cause prolonged defects in humoral immunity, as demonstrated in mice (Pötschke et al., 2013). However, previous studies on impaired antibody responses have focused on evaluation of vaccine efficacy rather than natural immunization by IPD. Prior carriage of *S. pneumoniae* in the nasopharynx, as well as prior bacteremia has been associated with a hyporesponse to PCV and PPV23, respectively (Ekdahl et al., 1997b; Dagan et al., 2010a). Ekdahl and collaborators suggested that IgG deficiency caused the poor antibody response in patients with prior IPD when immunized with PPV23. We excluded IgG deficiency as the cause of a nonfunctional antibody response in our study.

The high rate of a nonfunctional antibody response in the present study contrasts to the low rate of recurrent IPD in epidemiological studies. However, nasopharyngeal carriage rates of pneumococci are low in the adult population, even for low invasive serotypes (Regev-Yochay et al., 2004). The risk for IPD with the same serotype at two occasions is therefore theoretically very low. King et al. (2003) suggested that host factors of immunosuppression and underlying illness were typical in patients with IPD reinfection, whereas only two serotypes (6B and 18C) were associated with reinfection . It cannot be excluded, however, that a nonfunctional antibody response induced by IPD resolves over time. In a recent vaccine trial, the hyporesponse induced in toddlers by a combined schedule of PCV7 and PPV23 was not sustained when the children were in preschool age (Licciardi et al., 2016).

In the present study, serum concentrations of serotype specific IgG were determined, and a ratio between post and pre-IPD serum was calculated (Figure 2). Patients with a functional antibody response had higher IgG titer ratios, which may indicate that an antibody increase is necessary for efficient pneumococcal killing. Nevertheless, a divergence in IgG ratios and results obtained by OPA was found in several cases. These findings are also supported by a Japanese study, where infants immunized with PCV7 demonstrated protective IgG titers post-IPD, but all 17 patients had suboptimal responses in OPA. Low avidity of serotype-specific antibodies was suggested as the cause (Oishi et al., 2013). We tested the hypothesis that lower avidity of anti-capsular IgG antibodies post-IPD may contribute to the discrepancy in some patients that were nonresponsive in OPA in spite of a high IgG-ratio. We did not, however, find any changed avidity that could explain the discrepancies observed with high IgG ratios and a nonresponsive opsonophagocytosis (data not shown). Another possible explanation for diverging results between the serum tested in OPA and IgG ratios may be differences in levels of anti-pneumococcal polysaccharide IgM levels. Interestingly, Park and Nahm (2011) found that low levels of IgM in older adults contributed to a poor opsonization of pneumococci.

A limitation of this study was that we only included seven of more than 90 known pneumococcal serotypes. The rationale for this was that these sera were the only specimens from patients with IPD identified in our biobanks during the study period. From a clinical point of view, however, these 7 serotypes are considered to be among the most important ones (Hausdorff et al., 2000). Furthermore, serum samples were collected with a great variation in time prior to and after the episode of IPD that has to be considered when interpreting the results of this study. Therefore, we address the importance of conducting a prospective study where the collection pre- and post-IPD sera is defined as per protocol. Finally, we did not collect any vaccination data in this study. Yet, it has to be noticed that the usage of the 23-valent pneumococcal polysaccharide vaccine, which is recommended for adults, was very low in Sweden during the study period (only 0.08 prescriptions per 1,000 adults) (The Board of Health and Welfare, 2018).

## Conclusion

A nonfunctional antibody response may follow an episode of IPD. The risk of a poor humoral response was in our material associated to old age and infection with a pneumococcal serotype with low invasive potential.

## Materials and Methods

### Patient Sera

Patient characteristics are outlined in the Supplementary Material. Pre/acute-IPD and post-IPD sera used in this exploratory study were convenience samples that had been collected previously. Fourteen cases were obtained from a cohort at Örebro University Hospital and were collected in 1999–2002, and 26 cases from Skåne University Hospital (Biobank Skåne, Lund) in 2006–2017. No statistically significant differences in age, Charlsons Comorbidity Index or gender distribution were found between the cohorts. Amongst the 40 patients, paired sera from 20 patients were collected in the acute phase of IPD at admission to hospital and after 5 weeks of convalescence (median 34 days; range 20–81 days). For another 20 patients, paired sera were collected from patients 2–24 months prior to (pre-IPD; median 12 months) and 6–60 months after an episode of IPD (post-IPD; median 26 months) (Supplementary Material). A pre-IPD or acute-IPD serum is designated as “pre-IPD” in the study.

Based upon the study by Brueggemann et al. (2004), we sorted pneumococcal serotypes into two groups according to invasive potential. Twenty paired sera (pre-IPD and post-IPD) from patients infected by low invasive serotypes were included (serotype 3, *n* = 12; 19F, *n* = 4; 23F, *n* = 4), and for comparison, 20 paired sera from patients infected by highly invasive serotypes (serotype 7F, *n* = 11; 14, *n* = 5; 1, *n* = 2; 4, *n* = 2) were also added to the analyses. The medical history of all patients was reviewed. Disease severity of IPD was determined according to SIRS criteria (Bone et al., 1992), and comorbidities were defined according to the Charlsons Comorbidity Index (Charlson et al., 1987).

### Pneumococcal Serotyping

Blood for culture was collected at admission to hospital from all patients. Bactec^TM^ blood culturing system (Becton Dickinson, MD, United States) was used for isolation, and pneumococcal isolates were grown on 10% blood agar plates at 37° CO_2_ overnight. The Quellung test was performed for serotyping with specific rabbit antisera (Statens Serum Institut [SSI], Copenhagen, Denmark) (Habib et al., 2014). *S. pneumoniae* serotype 1, 3, 4, 7F, 19F, and 23F (control isolates from BEI resources, Manassas, VA, United States) were used in the OPA (Romero-Steiner et al., 1997).

### Enzyme-Linked Immunosorbent Assay (ELISA) for Determination of Capsular Antigen IgG

Homologous IgG antibody titers were quantified by ELISA according to a protocol from the World Health Organization (WHO) (Slotved et al., 2009). Serotype 22F specific capsular polysaccharide and cell-wall polysaccharide (both from SSI) were used for blocking of unspecific binding, and 007sp [kindly provided by Dr. Mustafa Akkoyunlu at U.S. Food and Drug Administration (FDA), Silver Spring, MD, United States] was used as reference serum. A ratio between pre-/acute-IPD and post-IPD was calculated for each patient.

### Opsonophagocytic Assay (OPA)

The functionality of antibody titers were determined by a single serotype OPA according to a protocol available at https://www.vaccine.uab.edu/uploads/mdocs/cdc-ops3.pdf (accessed 26/06/2018). The human promyelocytic cell-line HL60 was differentiated into neutrophils by propagation with addition of 0.8% dimethylformamide (DMF) for 5–6 days before use in the OPA. To prevent bacterial clumping, the opsonization and phagocytosis steps were performed with microtitre plates on a mini orbital shaker (700 rpm) as specified in a recent multiplex OPA protocol (WHO reference laboratory at University of Alabama). Patient sera were tested in at least two separate experiments in duplicates against the homologous serotype that had caused IPD. OPA titers were defined as the serum dilution that killed 50% of bacteria compared to controls containing no patient serum but cells only. A minimum titer of 1:8 in OPA has been shown to confer protection in mice and infants, and was therefore selected as cut-off (Romero-Steiner et al., 2006). Pre-IPD and post-IPD sera were tested for all patients. An increase in pneumococcal killing or unchanged OPA titer above the 1:8 titer threshold was termed functional antibody response and a decrease in OPA titer was termed decreased antibody response. OPA titers below the threshold before and after IPD were designated as a nonresponse. To test sera and exclude a residual antibiotic activity, a previously published method was used (Driscoll et al., 2012) with some modifications. Agar susceptibility testing was performed by adding sera to holes (2 mm in diameter) on blood agar plates inoculated with respective target strain, the same ones used in the OPA. After overnight culture, absence of a zone of bacterial killing indicated no antibiotic activity.

### ELISA for Determination of Total IgG and IgG2

Maxisorp^TM^ plates (Nunc, Waltham, MA, United States) were coated with rabbit anti-human IgG (Sigma, Darmstadt, Germany) or mouse anti-human IgG2 (Sigma) overnight at +4°C. Plates were washed in wash buffer (PBS pH 7.4, 0.05% Tween20), blocked with blocking buffer (PBS, 1% skimmed milk, 0.05% Tween20) for 1 h at room temperature (RT) followed by an additional wash. Patient and calibration sera for IgG (Dako, Glostrup, Denmark) and pure IgG2 (The Binding site, San Diego, CA, United States) were diluted in a blocking buffer, titrated on plates and incubated for 1 h at RT. After one final wash, all wells were incubated for 20 min with horseradish peroxidase (HRP)-conjugated rabbit anti-human IgG (Dako P0214). The optical density was measured at 405 nm, and absorbance between calibration sera and patient sera was compared.

### Statistical Analyses

Three groups (increased antibody response, nonresponse and decreased antibody response) were compared using the Chi-squared test for categorical variables and the Kruskal-Wallis test for continuous variables. Univariate logistic regressions were performed to assess the association between the type of antibody response and clinical as well as bacterial predictors. In all regression analyses, the two types of nonfunctional responses (nonresponse or decreased response) were collapsed to create a binary outcome variable. A multivariate model was fitted using the purposeful selection algorithm. Briefly, all variables with a *p*-value below 0.2 were added to the multivariate model. The variable with the highest *p*-value was stepwise removed and the model was run again until only significant variables or variables that affected the adjusted odds ratios or *p*-values of the remaining variables remained. Comparisons of difference between the two groups in Figure 1B was made by Fisher’s exact test and in Figure 2 by Mann-Whitney *U* test. Statistical analyses were made in the SPSS^®^ v.22 software or in Prism Graphpad^®^ 7 and a level of significance was set to *p* ≤ 0.05.

## Data Availabilty Statement

We will make materials, data, and associated protocols promptly available to readers upon request.

## Ethics Statement

This study was approved by the Regional Ethics Board at Lund University Hospital (2012/86) and the Ethics committee of the Örebro County Council (868–1999). The methods were done in accordance with the Helsinki declaration and regulations of the abovementioned Universities and Biobank Skåne.

## Author Contributions

NL designed the study, analyzed the data, performed the experiments and drafted the manuscript. FU did the experiments and contributed to the writing. JA designed and initiated the study and revised the manuscript. FR helped in designing the study, performed the statistical analysis and revised the manuscript. H-CS contributed to the design of the study and critically revised the manuscript. SA analyzed the patient data, collected the patient sera and critically revised the manuscript. KR initiated and designed the study and critically revised the manuscript. All authors have approved the final manuscript.

## Conflict of Interest Statement

H-CS, JA, and KR are participating in other projects supported by Pfizer. KR has been collaborating with GSK and been a scientific advisor to MSD. JA received payments for lectures from Astrazeneca, GSK, MEDA and Pfizer. The remaining authors declare that the research was conducted in the absence of any commercial or financial relationships that could be construed as a potential conflict of interest.
