# Supplementary material for: Serotypes With Low Invasive Potential Are Associated With an Impaired Antibody Response in Invasive Pneumococcal Disease
Source: Front Microbiol. 2018 Nov 15;9:2746. doi: 10.3389/fmicb.2018.02746 (PMC6249558; doi:10.3389/fmicb.2018.02746)
Supplement: Supplementary file 1 [file Table_1.docx]

**Supplementary Table S1**

**Serotypes with low invasive potential are associated with an impaired antibody response in invasive pneumococcal disease**

Nils Littorin *et al.*

Table S1. Clinical data on all patients included in the present study.

| **Patient ID** | **Serotype** | **Invasive potential** | **Sex** | **Age** | **Charltons comorbidity index** | **SIRS category** | **Period of serum collection post-IPD (months)** | **Anti capsular IgG pre/acute (mg/L)** | **Anti capsular IgG convalescent (mg/L)** | **Post- /pre IPD IgG titer ratio** | **Functional antibody response in OPA** |
| --- | --- | --- | --- | --- | --- | --- | --- | --- | --- | --- | --- |
| 1 | 1 | **High** | F | 61 | 2 | Severe | 1 | 3.10 | 3.26 | 1.05 | Nonresponse |
| 2 | 1 | **High** | F | 32 | 0 | Severe | 31 | 4.30 | 4.25 | 0.99 | Functional response |
| 3 | 3 | **Low** | M | 59 | 2 | Severe | 1 | 0.19 | 11.6 | 61.1 | Nonresponse |
| 4 | 3 | **Low** | M | 48 | 0 | Sepsis | 1 | 0.73 | 1.50 | 2.0 | Decreased response |
| 5 | 3 | **Low** | M | 78 | 7 | Chock | 60 | 0.63 | 0.05 | 0.08 | Nonresponse |
| 6 | 3 | **Low** | F | 55 | 1 | Severe | 30 | 0.18 | 0.36 | 2.00 | Nonresponse |
| 7 | 3 | **Low** | F | 53 | 1 | Missing | 26 | 0.40 | 0.73 | 1.81 | Functional response |
| 8 | 3 | **Low** | F | 61 | 2 | Severe | 35 | 0.18 | 0.30 | 1.67 | Nonresponse |
| 9 | 3 | **Low** | M | 70 | 6 | Severe | 38 | 0.09 | 0.05 | 0.50 | Nonresponse |
| 10 | 3 | **Low** | F | 46 | 0 | Severe | 25 | 0.75 | 0.1 | 0.13 | Decreased response |
| 11 | 3 | **Low** | F | 56 | 4 | Chock | 1 | 0.69 | 1.45 | 2.10 | Functional response |
| 12 | 3 | **Low** | M | 60 | 1 | Severe | 10 | 0.73 | 1.31 | 1.8 | Functional response |
| 13 | 3 | **Low** | M | 54 | 3 | Chock | 1 | 5.50 | 14.52 | 2.64 | Nonresponse |
| 14 | 3 | **Low** | F | 54 | 1 | Severe | 1 | 1.79 | 5.63 | 3.15 | Nonresponse |
| 15 | 4 | **High** | M | 87 | 7 | Severe | 1 | 0.55 | 0.67 | 1.21 | Nonresponse |
| 16 | 4 | **High** | F | 75 | 3 | Sepsis | 1 | 0.70 | 6.67 | 9.50 | Functional response |
| 17 | 7F | **High** | F | 89 | 5 | Sepsis | 1 | 8.90 | 7.03 | 0.79 | Functional response |
| 18 | 7F | **High** | F | 67 | 2 | Sepsis | 1 | 10.30 | 28.70 | 2.87 | Functional response |
| 19 | 7F | **High** | F | 57 | 1 | Sepsis | 1 | 15.01 | 4.95 | 0.33 | Decreased response |
| 20 | 7F | **High** | M | 39 | 3 | Severe | 1 | 3.45 | 8.32 | 2.4 | Functional response |
| 21 | 7F | **High** | M | 31 | 0 | Sepsis | 1 | 2.00 | 11.76 | 5.88 | Functional response |
| 22 | 7F | **High** | F | 28 | 0 | Chock | 39 | 1.10 | 2.1 | 1.90 | Nonresponse |
| 23 | 7F | **High** | F | 67 | 8 | Sepsis | 19 | 0.62 | 1.7 | 2.70 | Functional response |
| 24 | 7F | **High** | M | 55 | 1 | Sepsis | 60 | 0.60 | 9.1 | 15.20 | Nonresponse |
| 25 | 7F | **High** | F | 82 | 6 | Chock | 24 | 0.52 | 1.03 | 1.98 | Nonresponse |
| 26 | 7F | **High** | F | 25 | 0 | Sepsis | 23 | 1.03 | 4.15 | 4.03 | Functional response |
| 27 | 7F | **High** | M | 38 | 0 | Chock | 1 | 0.70 | 8.30 | 11.90 | Functional response |
| 28 | 14 | **High** | F | 47 | 0 | Sepsis | 0.5 | 7.11 | 38.2 | 5.40 | Functional response |
| 29 | 14 | **High** | M | 67 | 4 | Severe | 25 | 4.75 | 2.2 | 0.46 | Nonresponse |
| 30 | 14 | **High** | M | 64 | 4 | Sepsis | 6 | 4.74 | 9.1 | 1.93 | Functional response |
| 31 | 14 | **High** | M | 40 | 1 | Chock | 12 | 37.2 | 4.75 | 0.13 | Functional response |
| 32 | 14 | **High** | F | 91 | 4 | Sepsis | 1 | 5.96 | 1.07 | 0.18 | Decreased response |
| 33 | 19F | **Low** | F | 83 | 4 | Severe | 1 | 8.30 | 5.81 | 0.70 | Decreased response |
| 34 | 19F | **Low** | M | 63 | 4 | Chock | 11 | 8.47 | 14.61 | 1.72 | Decreased response |
| 35 | 19F | **Low** | F | 24 | 0 | Sepsis | 55 | 8.77 | 6.1 | 0.69 | Functional response |
| 36 | 19F | **Low** | M | 14 | 6 | Sepsis | 52 | 14.7 | 7.3 | 0.50 | Nonresponse |
| 37 | 23F | **Low** | M | 85 | 4 | Severe | 1 | 2.80 | 9 | 2.73 | Nonresponse |
| 38 | 23F | **Low** | F | 85 | 6 | Sepsis | 1 | 3.00 | 6.96 | 2.32 | Nonresponse |
| 39 | 23F | **Low** | F | 84 | 4 | Sepsis | 1 | 22.40 | 17.92 | 0.88 | Nonresponse |
| 40 | 23F | **Low** | M | 70 | 4 | Severe | 1 | 1.10 | 2 | 1.45 | Nonresponse |
